# Organization of rehabilitation care in Portuguese intensive care units

**DOI:** 10.5935/0103-507X.20180011

**Published:** 2018

**Authors:** Roberto Miguel Gonçalves Mendes, Manuel Lourenço Nunes, José António Pinho, Ricardo Bruno Rodrigues Gonçalves

**Affiliations:** 1 Faculdade de Ciências da Saúde, Universidade da Beira Interior - Covilhã, Portugal.; 2 Intensive Care Unit, Unidade Local de Saúde de Castelo Branco - Castelo Branco, Portugal.; 3 Intensive Care Service 1, Centro Hospitalar do Porto - Porto, Portugal.; 4 Intensive Care Unit, Serviço de Saúde da Região Autónoma da Madeira - Funchal, Portugal.

**Keywords:** Critical care, Rehabilitation nursing, Hospital physical therapy service/organization & administration, Portugal

## Abstract

**Objective:**

To describe the different rehabilitation care models in practice in Portuguese adult intensive care units.

**Methods:**

A simple observational (cross-sectional) study was conducted through an online survey sent to the head nurses or individuals responsible for the 58 adult intensive care units that are part of the database of the *Sociedade Portuguesa de Cuidados Intensivos*.

**Results:**

We identified three models of organization of rehabilitation care: care provided by the staff of the intensive care unit (22.9%), care provided by specialized external teams (25.0%), and a mixture of the previous models, combining the two situations (52.1%). In the first model, the care was provided mainly by nurses with specialization in rehabilitation and, in the second model, the care was provided by physiotherapists. No significant differences were found between the models regarding the availability of care, in hours/day or days/week (p = 0.268 and 0.994, respectively), or results such as length of hospital stay in intensive care, ventilation time, or mortality rate in the unit (p = 0.418, 0.923, and 0.240, respectively).

**Conclusion:**

The organization of rehabilitation care in Portuguese intensive care units is unique and heterogeneous. Despite different care organization models, the availability of hours of care is similar, as are the overall results observed in patients.

## INTRODUCTION

In intensive care units (ICU), the known effects of prolonged immobilization are enhanced by the development of neuropathy or myopathy resulting from the disease itself.^(1-3)^ Even after overcoming the acute phase, patients often undergo states of great physical/functional impairment and are sometimes unable to perform simple daily life activities, and psychosocial impairment, which compromises social and professional reintegration. All of these findings are associated with a reduction in the quality of life below the average of the general population.

The bed rest theory is already part of the past; early rehabilitation is a safe and beneficial practice. According to a meta-analysis performed in 2014,^(3)^ there is evidence that this practice, together with glycemic control, plays a protective role in the development of neuromuscular disorders arising from critical illness.

Although there are international recommendations on ICU rehabilitation^(4-6)^ and more or less comprehensive early mobilization protocols are being disseminated,^(7-9)^ the Portuguese reality is little known. Moreover, the Portuguese context, besides different hospital management models, has particularities in the organization of the rehabilitation care itself, with a multiplicity of scenarios, without knowing the work developed in each center or its results.

The primary objective of this research was to describe the different models of rehabilitation care in practice in the Portuguese adult ICUs. The secondary objectives were to quantify the number of professionals with training in rehabilitation available in each unit, to verify the providers and prescribers of rehabilitation care in each model, and to identify the model that guarantees more hours of care and better results.

## METHODS

A simple observational (cross-sectional) study was conducted through an *online* survey directed to head nurses or individuals responsible for the adult ICU, levels II and III, who were part of the database of the *Sociedade Portuguesa de Cuidados Intensivos* (SPCI), with approval by the Ethics Committee of the *Universidade da Beira Interior* (Opinion EC-FCS-2016-028).

The survey consisted of 28 questions, grouped into the following categories: characterization of the institution, which identified the management model and the classification of the institution, taking into account the nature of their responsibilities and the capacity chart (Ordinance 82/2014);^(10)^ characterization of the unit, identifying its type and the number of active beds; characterization of the team, quantifying the number of professionals from different care areas, distinguishing those who worked full-time from those who worked part-time; and rehabilitation care organization, which identified the care organization model and the providers and their forms of planning and implementation. The following was also verified: existence of functional evaluation at discharge, follow-up after discharge, and use of indicators related to rehabilitation practices, availability of Human Resources (in terms of hours and days of available care) and material resources for rehabilitation. Regarding to the last year, were asked: number of patients admitted, mean severity, ICU length of stay, mean duration of invasive ventilation, and mortality rate in the unit.

This questionnaire was prepared by the team of researchers and was reviewed by experts of the *Associação Portuguesa de Fisioterapeutas* and the *Associação Portuguesa dos Enfermeiros Especializados em Enfermagem de Reabilitação*. A nurse specialized in rehabilitation nursing, with leadership roles, and two physical therapists working in an ICU also checked the questions.

Data collection took place from November 1, 2016 to March 1, 2017.

Statistical analysis was performed using the IBM *Statistical Package for Social Sciences* (SPSS), version 22. Descriptive statistics were calculated by means of frequencies, percentages, means, and standard deviations. The analysis of the independence of the care organization model in relation to the institutional management model, the degree of hospital differentiation, and the ICU classification was performed using the Pearson chi square test obtained by Monte Carlo simulation. The comparison of the different models in terms of hours/days of care, number of patients admitted, and their severity was performed using the Kruskal-Wallis test. The comparisons of the care results, hospitalization time, ventilation time, and mortality rate were performed using analysis of variance (ANOVA). A significance level of 0.05 was used.

## RESULTS

Surveys were sent to head nurses or individuals responsible for the 58 ICUs belonging to 51 hospitals. A total of 54 surveys were answered, 6 of which were excluded because less than two-thirds of the answers were valid, totaling 48 valid surveys. The high completion rate of this survey was due to, in part, the relevance of the subject and also the methodology used: the survey was sent after the first telephone contact, which the aim of introducing the researcher and the objectives of the research.

The sample obtained included mostly ICUs integrated in Group I (less differentiated) hospitals. The management model of these institutions was predominantly the business public, and the units were mainly medical-surgical or polyvalent units (Table 1). This sample represented a total of 399 intensive care beds and 132 intermediate care beds (corresponding to 18 units that formed intermediate care services).

**Table 1:** Characterization of the participating intensive care units

Characteristics	n (%)
Institutional classification	
Group I hospital	25 (52.1)
Group II hospital	8 (16.7)
Group III hospital	14 (29.2)
Did not answer	1 (2.1)
Institutional management model	
Corporate public entity	43 (89.6)
Public-private partnership	4 (8.3)
Did not answer	1 (2.1)
ICU classification	
Medical-surgical	28 (58.3)
Cardiothoracic	2 (4.2)
Neurosurgery	3 (6.3)
Other (multipurpose)	14 (29.2)
Did not answer	1 (2.1)

ICU - intensive care unit.

### Multidisciplinary team

Nurses constituted the majority professional class, followed by doctors. Statistically, approximately one in ten (9.4%) nurses had specialized training in rehabilitation nursing; 92% of Portuguese ICUs had nurses with this specialization, although they performed specialized functions in only 75% of them. Approximately 46% of the units had rehabilitation nurses performing full-time specialized functions, 29% had only part-time rehabilitation nurses, and 25% did not have a rehabilitation nurse in functions. Only three physical therapists worked full time in ICUs, and speech therapists or occupational therapists worked just occasionally and in part-time.

### Organization of care

**Figure 1:**
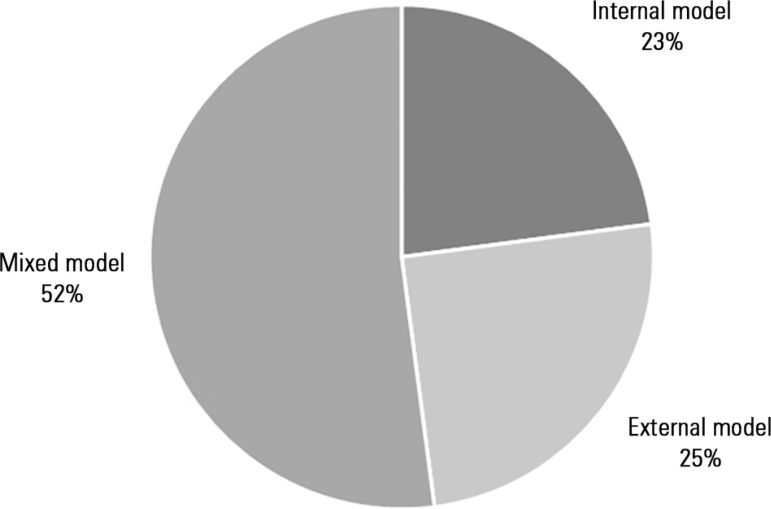
Models of rehabilitation care organization.

Three models of rehabilitation care organization were identified (Figure 1): an internal model, where the care was performed by the ICU's own team (22.9%); an external model, in which care was provided by a specialized team external to the ICU (25.0%); and a mixed model, in which care was provided by the ICU team in conjunction with a specialized external team (52.1%).

By crossing the distribution of these models of care with the institutional management model, it was observed that both corporate public entities and public-private partnerships dominated the mixed care model (51% in corporate public entities and 75% in public-private partnerships). However, inferential analysis allowed us to state that the organization of rehabilitation care was independent of the institutional management model (X^2^_(2)_ = 1.419, p = 0.797, N = 43).

By performing a similar analysis for the ICU classification, the mixed model was found to also predominate in the medical-surgical units (43%), polyvalent units (57%), and neurosurgical units (100%). In the cardiothoracic units, the mixed and internal models represented the same proportion (50%). However, the organization of rehabilitation care was also independent of the ICU classification (X^2^_(6)_ = 6.498, p = 0.370, N = 47).

Regarding the degree of hospital differentiation, the mixed model (48% and 79%) predominated in Group I and III hospitals, and the specialized external model (63%) predominated in Group II, with statistically significant differences (X^2^_(4)_ = 12.178, p = 0.015, N = 47).

Rehabilitation care in Portuguese ICUs was provided by several professionals, with emphasis on physical therapists and nurses who are specialists in rehabilitation nursing, as they have a more frequent participation. In the internal care model, the providers were mostly nurses with a specialty in rehabilitation nursing, popularly designated as rehabilitation nurses (all units with an internal model comprised rehabilitation nurses). In the case of care provided by a specialized external team, the providers were mainly physical therapists (67.7%), followed by rehabilitation nurses (18.9%).

**Figure 2:**
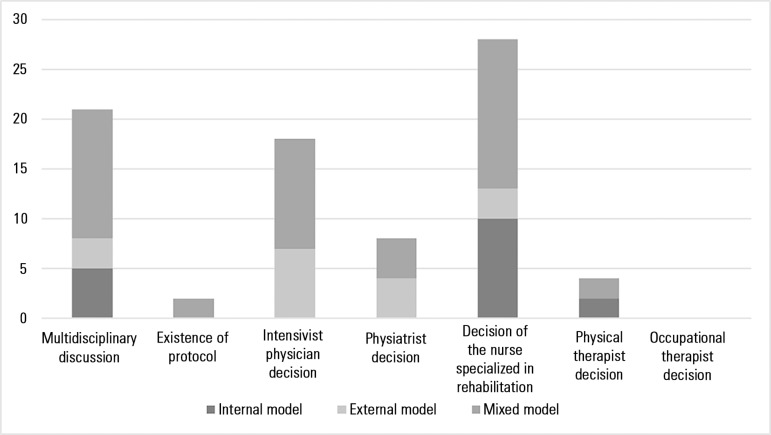
Decision making for starting rehabilitation care.

**Figure 3:**
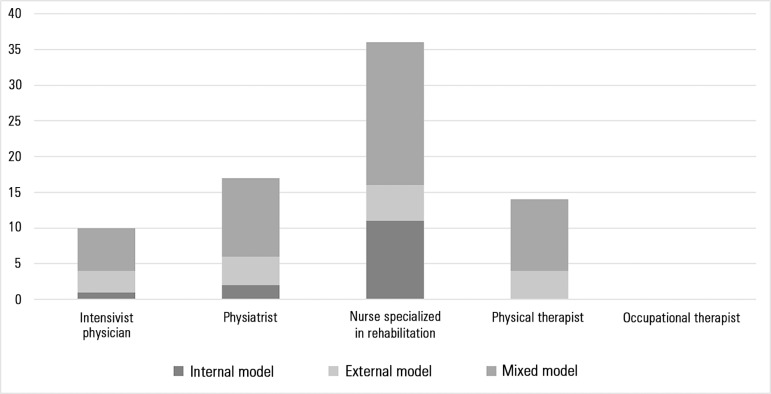
Elaboration of the rehabilitation program.

The decision to start rehabilitation in a severely ill patient was taken more often by rehabilitation nurses, whether unilaterally or by multidisciplinary team discussion. In the model of care provided by a specialized external team, this role was mainly performed by the intensivist physician (Figure 2). In addition, for the preparation of the rehabilitation care plan, the role of rehabilitation nurses, regardless of the care organization model, was highlighted (Figure 3).

For the evaluation of patients at discharge, functional aspects were assessed in nine of the units (22.0%). The model of rehabilitation care was mixed in five of the units, internal in three units, and external in one unit.

Evaluation after discharge was performed in six units (12.5%), and the involvement of a physical therapist was reported only once in this assessment, which was made mainly by the physician and nurse. Two ICUs from each model of care organization evaluated patients after discharge.

Indicators related to rehabilitation practices were obtained in ten units (20.8%), mostly consisting of the group in which the organization of rehabilitation care was performed according to the mixed model (six units with a mixed model and two with an internal model).

### Availability of resources

Regardless of the model of care organization and the time each professional dedicated to the ICU, 77.1% of the units had rehabilitation nursing care, 68.8% had physical therapy, 14.6% had occupational therapy, and 8.3% had speech therapists. On average, rehabilitation care was available 5.83 ± 4.24 hours/day and 5.02 ± 2.17 days/week. Although the ICU internal team model assures more hours of care per day and more days per week, these differences were not statistically significant (Table 2).

**Table 2:** Availability of rehabilitation care

Variable	Internal model (n = 11)	External model (n = 12)	Mixed model (n = 25)	p value
Hours of care/day	7.18 ± 5.21	4.17 ± 3.10	6.04 ± 4.14	0.268
Days of care/week	5.27 ± 1.49	4.92 ± 2.47	4.88 ± 2.34	0.994

Only 39.58% of the units presented their results in terms of length of stay in the ICU, time of invasive ventilation, and mortality rate in the ICU. The mean time of hospitalization was 7.38 ± 2.25 days, the time of invasive ventilation was 5.73 ± 2.69 days, and the mortality rate was 20.80 ± 6.07%. These results were independent of the model of rehabilitation care organization (Table 3).

**Table 3:** Health care results in the last year

Indicator	Internal model	External model	Mixed model	p value
Accepted patients	320.00 ± 112.59	258.00 ± 186.82	389.50 ± 230.64	0.297
Severity				
SAPS II	46.94 ± 2.91	27.00 ± 24.02	47.21 ± 5.73	0.128
APACHE II	20.57 ± 13.54	21.00	30.45 ± 12.19	0.156
Days of ICU stay	7.26 ± 2.37	8.13 ± 1.53	7.06 ± 2.53	0.418
Days of invasive ventilation	5.65 ± 3.70	6.10 ± 1.27	5.71 ± 2.47	0.923
Mortality in the ICU	24.29 ± 4.26	18.83 ± 7.14	19.52 ± 6.22	0.240

SAPS - Simplified Acute Physiology Score; APACHE II - Acute Physiology and Chronic Health Evaluation; ICU - intensive care unit.

## DISCUSSION

Although there are publications dealing with the use of certain rehabilitation techniques in critical patients or with the benefits of rehabilitation in general, the organization of rehabilitation care in ICUs is not well known. At the European level, in 2000, the profile of physical therapy in ICUs was published.^(11)^ According to this study, 75% of the units had exclusive physical therapists, and the results for Portugal (at that time represented by seven ICUs) were in agreement with those in the rest of Europe. In the United States, in 2015, 34% of ICUs had a dedicated physical therapist and/or occupational therapist,^(12)^ and in Japan, in 2016, 77% of ICUs had rehabilitation care on-call regimes.^(13)^

Although Portugal participated in the European analysis 17 years ago, we had the perception that, at present, the results could be divergent, not only because of the period of time that had elapsed, but also because of the development observed in the organization of the units, the specialized training of physical therapists, in the area of intensive care, and intensive care nurses, in rehabilitation nursing. Other studies that have indirectly addressed this issue focused on the intervention by physical therapists^(14)^ or on the allocation of rehabilitation nurses.^(15)^ Both professional classes are relevant in this context: rehabilitation nursing was normally integrated into the ICU team, and physical therapy was generally integrated into physical medicine and rehabilitation services. In most units, regardless of type or management model, the most frequent scenario is the articulation of these two situations. Despite the different forms of organization, the availability of hours of care is similar, and the overall results are also similar. It would be interesting to analyze results more sensitive to rehabilitation care, but the number of units using specific indicators is still low, and these indicators are relatively heterogeneous; therefore, they were not included in this analysis.

As in 2000,^(11)^ rehabilitation care at night is not yet available. Nevertheless, 16.7% of the units offer rehabilitation care for more than 8 hours a day, in contrast to 10.4% who reported zero hours/day, suggesting that this type of care is not part of their daily practice. In addition, only one unit reported that rehabilitation was conducted according to existing protocols. These results can be improved if we consider that early rehabilitation of the critically ill patients is safe and beneficial and that the systematization of care through protocols shows clear benefits.^(9,16-18)^

We conclude this analysis by pointing out the number of nurses with specialized training in rehabilitation (approximately 10%) to integrate the ICU teams, even though some services do not perform functions in the area. The intervention role of these professionals is emphasized not only in the direct provision of care but also in the planning of care. The presence of these professionals in the rehabilitation care organization of the critically ill patient makes Portugal a particular case, justifying this individual analysis.

This study has potential limitations. We attempted to minimize the bias by inquiring of all the national ICUs that were part of the SPCI database, and the participation rate was quite positive (82.76%). To avoid the possibility of receiving more than one response from the same respondent, we blocked the user after one response (not the IP, because it could be the same in units at the same institution). To stimulate participation, we reduced the size of the survey, choosing not to include questions to characterize the profiles of respondents or rehabilitation elements. To collect as much information as possible, we allowed blank responses and, afterwards, all surveys with more than two-thirds of valid answers were selected. There may have been some bias for just asking the head nurse or the person in charge and for not questioning elements from other areas of expertise. This option was chosen because we consider that within the multidisciplinary team and taking into account the various specificities related to this topic, the head nurse is a figure present in all contexts and is able to respond to different issues.

Future work should seek to characterize the rehabilitation practices of each unit and to compare results with each other and with other countries where the realities of rehabilitation are different.

## CONCLUSION

The Portuguese reality was singular and heterogeneous. We identified an internal organization model, provided mainly by rehabilitation nurses; an external model, mostly provided by physical therapists; and a mixed model, usually involved the participation of both. Despite the different models of care organization, the availability of hours of care was similar. However, the analysis of results and the obtaining of indicators sensitive to rehabilitation care were, in most cases, marginal aspects to the different models. Yet, the available results did not show differences between the models.
